# Characterization of Viral Load, Viability and Persistence of Influenza A Virus in Air and on Surfaces of Swine Production Facilities

**DOI:** 10.1371/journal.pone.0146616

**Published:** 2016-01-12

**Authors:** Victor Neira, Peter Rabinowitz, Aaron Rendahl, Blanca Paccha, Shawn G. Gibbs, Montserrat Torremorell

**Affiliations:** 1 University of Minnesota, Saint Paul, Minnesota, United States of America; 2 University of Chile, Santiago, Chile; 3 University of Washington, Seattle, Washington, United States of America; 4 Yale University, New Haven, Connecticut, United States of America; 5 Indiana University, Bloomington, Indiana, United States of America; Virginia Polytechnic Institute and State University, UNITED STATES

## Abstract

Indirect transmission of influenza A virus (IAV) in swine is poorly understood and information is lacking on levels of environmental exposure encountered by swine and people during outbreaks of IAV in swine barns. We characterized viral load, viability and persistence of IAV in air and on surfaces during outbreaks in swine barns. IAV was detected in pigs, air and surfaces from five confirmed outbreaks with 48% (47/98) of oral fluid, 38% (32/84) of pen railing and 43% (35/82) of indoor air samples testing positive by IAV RT-PCR. IAV was isolated from air and oral fluids yielding a mixture of subtypes (H1N1, H1N2 and H3N2). Detection of IAV RNA from air was sustained during the outbreaks with maximum levels estimated between 7 and 11 days from reported onset. Our results indicate that during outbreaks of IAV in swine, aerosols and surfaces in barns contain significant levels of IAV potentially representing an exposure hazard to both swine and people.

## Introduction

Influenza A virus (IAV) causes significant epidemics of respiratory disease in humans that result in human deaths and raise public health concerns that require a deeper understanding of IAV epidemiology and control. IAV is shared among animals and people and novel viruses capable of causing pandemics are the result of reassortant viruses from different species. Despite evidence that reassortment can happen in various species, swine is often labeled as the “mixing vessel” since swine have receptors capable to replicate influenza viruses of avian, human and swine origin. Because these viruses can infect humans, understanding transmission of swine-origin IAVs should be a priority.

In addition to IAV being a major pathogen for humans, IAV is also a serious problem in swine causing frequent outbreaks that involve both animal illness and zoonotic infections [1–3]. In swine, IAV is distributed worldwide and is endemic in the US swine herd [2]. For almost a century, classical H1N1 viruses were the dominant IAVs until the appearance and subsequent circulation of double and triple reassortants since 1998 [4–6]. More recently, the 2009 pandemic virus [7], and the on-going influx of human-origin IAVs in swine [8, 9] has led to a more complex epidemiologic picture, making control of influenza in swine very difficult. The 2009 H1N1 pandemic, as well as outbreaks of variant H3N2 (H3N2v) influenza have demonstrated the potential for swine origin IAVs to cause significant morbidity and mortality globally, impacting the general public, swine workers and animal agriculture [10, 11]. Swine workers in particular, and their non-swine-exposed spouses, have been shown to be at a higher risk of swine-origin IAV infections than the general public [12], leading to calls for including such workers in pandemic preparedness and surveillance [13]. Since both direct and indirect contact exposures in commercial swine and agricultural fairs have been suspected in IAV zoonotic infections [10, 13], influenza prevention efforts involving swine production need to address multiple potential exposure routes.

While it is known that transmission of IAV occurs by direct contact, IAV can also be transmitted through indirect routes. Transmission of IAV via contaminated personnel and fomites has been documented in pigs [14] and aerosol transmission of IAV has been reported in various species [15–21]. In swine, IAV has been detected in aerosols from immune swine [22–24] and more recently IAV was isolated from air samples from inside and outside swine farms [23], and live animal markets in Minnesota [3].

Despite the growing evidence of indirect transmission of swine-origin IAV, there is limited information on the natural dynamics of IAV outbreaks in swine environments including production facilities. Information is lacking on levels of exposure encountered by both swine and people exposed to swine aerosols or contaminated surfaces in swine facilities during outbreaks of IAV. Therefore, our objective was to characterize viral load, viability and persistence of IAV in the air and on surfaces during periods of active IAV outbreaks in swine production facilities. This knowledge would further our understanding of the risk of IAV transmission between swine and people, and help inform prevention efforts.

## Material and Methods

Procedures and protocols used in this study were approved by the University of Minnesota Institutional Animal Care and Use Committee protocol # 1207B17281 and the Institutional Biosafety Committee protocol # 1208H18341. Prior to the start of the study signed consent forms were obtained from the participating herds and forms were signed by herd owners or the production managers. No protected species were sampled.

### Farm identification and selection

Eleven investigations of IAV outbreaks in six swine farms were conducted from October 2012 to May 2013. Farms with suspected outbreaks were identified by contacting veterinarians in Southern Minnesota and Northern Iowa. Veterinarians were asked to alert the investigators upon sudden onset of respiratory clinical signs suggestive of acute influenza in a swine herd (i.e. rapid onset of widespread dry hacking cough, sneezing, rhinorrhea, anorexia and lethargy). Each investigation consisted of visiting a candidate farm multiple times to assess herd health, collect samples, and gather additional information including temperature and relative humidity data. Farms were included in the study if the veterinarian made a presumptive diagnosis of IAV infection in the herd or was able to collect samples and confirm the diagnosis within 4 days from the onset of clinical signs, and was able to communicate with the investigators within 3 days from the onset of disease.

The investigators visited the farm within 1 to 3 days of being contacted and the clinical history of the outbreak was reviewed after interviews with farm personnel. During each visit air samples from inside and outside, pig oral fluids and surface samples were collected. The number of visits in each investigation varied based on diagnostic results on samples from the prior visit. If a herd tested negative in oral fluids, the investigation for that herd was concluded in terms of additional visits for most of the cases. The number of visits per farm ranged between 1 and 10 and the longest outbreak was 42 days. A summary of the farm characteristics is shown in Table 1.

**Table 1:** Summary of farm characteristics.

Investigation ID	Farm ID	Barn ID	Days of sampling/ farm	Duration of outbreak (days)	Approximate No.pigs/ farm	Approximate No.pigs/ barn	Area of barn (m^2^)	Volume of barn (m^3^)	Pig age at infection onset (weeks)
1	1	1	2	4	2400	315	241	530	18
2	2	1	3	NA*	2400	1023	763	1860	8
3	3	1	4	NA	3400	1200	1106	2360	10
4	3	2	4	11	3400	1200	1106	2360	10
5	3	3	6	22	3400	1000	1106	2360	10
6	4	1	1	NA	3840	940	1508	3218	20
7	5	3	1	NA	2480	1000	821	1877	19
8	6	1	3	NA	12000	2010	892	2039	6
9	6	8	7	21	12000	2020	892	2039	7
10	6	2	8	28	12000	2006	892	2039	4
11	6	4	10	42^†^	12000	2010	892	2039	4

*NA: Non-applicable (investigation was IAV negative)

†: This investigation was considered two be two outbreaks

### Sampling procedures and sampling scheme

#### Oral fluids

Oral fluids were collected to determine whether IAV was present in the swine at the time of sampling. Swine oral fluids were sampled using ropes as described previously [25, 26]. Briefly, two 3-strand twisted cotton ropes (WebRiggingSupply.com, Barrington, IL 60010, USA) were placed in 2–4 pens for 20 min for the swine to chew on the ropes. Each rope was estimated to sample approximately between 20 to 50 swine depending on pen size. Oral fluids were extracted from the rope immediately after collection by wringing the wet portion into a plastic bag and then the fluid was transferred into a 5 ml plastic sterile tube, and samples refrigerated at 4°C until processing. Oral fluid samples were processed within 24 hours of collection, centrifuged for 10 min at 5,000 RPM, and stored at -80°C until tested by RRT-PCR (real time reverse transcriptase polymerase chain reaction) and virus isolation.

#### Air sampling

Upon arrival at the farm, the first set of air samples was collected outside the barn approximately 25 m upwind (n = 2). After that, the second set was collected downwind (n = 2) from the facility at approximately the same distance, and lastly the final set was collected in the barn interior (n = 2). For the air interior samples, air collectors were placed within the barn at approximately 1/3 and 2/3 the length of the building and 1.5 m above the floor. Each set of samples was collected simultaneously as duplicates. Swine did not have direct contact with the air collectors.

Air samples were collected using a liquid cyclonic collector (Midwest Micro-Tek, Brookings, SD, USA) capable of processing 200 L / min of air [23, 27]. Briefly, 10 mL of minimum essential medium (MEM) solution supplemented with 2% bovine serum albumin (BSA) were added to the collection vessel, and samples collected for 30 minutes. About 4 mL of collection media were recovered for each sample, media were transferred into a plastic vial with a syringe and stored on ice until transport within 12 hours to the laboratory.

#### Surface sampling

Surface samples were collected from areas considered to have high contact by humans working in the barns including pen railings (n = 2) and door handles from doors leading into the swine barns (n = 1). Surface samples were collected using a 2”x2” sterile gauze dipped into sterile MEM supplemented with 2% BSA. Sections of 1 m of pen railing with approximately 0.08 m^2^ (800 cm^2^) of surface, were wiped for 30 seconds using sterile gloves. Door handles were wiped for 15 seconds and both the exterior and interior handles were sampled. Pigs did not have direct contact with the pen railing as only the top railing was sampled. Gauzes were placed into individual tubes and samples stored on ice for transport and processing within 24 hours.

Oral fluids and surface samples were collected simultaneously at the same time that the air interior was being sampled.

### Diagnostic procedures

Oral fluid samples were first screened at the University of Minnesota Veterinary Diagnostic Laboratory for influenza A RNA by a RRT-PCR targeting the matrix gene [28]. Samples with a cycle threshold (ct) value <35 were considered positive, 35–40 suspect, and >40 negative. Samples with ct < 40 were further tested using a quantitative RRT-PCR test as described previously [23]. RRT-PCR positive samples were cultured for virus isolation using Madin-Darby Canine Kidney (MDCK) cells [29, 30] and subtyped using the Path-ID Multiplex One-Step RRT-PCR kit (Applied Biosystems, Foster City, CA, USA) and custom subtyping assay primers and probes (Life Technologies) [31].

### Swine clinical scores

Swine were visually inspected during each visit by a veterinarian member of the study team. Clinical scores consisted of coughing and sneezing and were measured following previously described procedures [32]. Briefly, the number of cough and sneeze episodes observed in 4 pens during 3 minutes were recorded. A cough or sneeze episode was defined as one or several coughs or sneezes in a sequence by an individual pig. The percentage of coughing or sneezing swine was calculated by dividing the number of swine observed coughing or sneezing by the total number of animals observed in the pens. The total number of swine evaluated in each visit ranged from 100 to 400 depending on pen and barn size.

### Environmental conditions

Temperature and relative humidity inside and outside the barns were recorded at the time of collection using a weather meter (Kerstrel 3000, Nielsen-Kellerman, PA, USA).

### Statistical and influenza modeling in indoor air and statistical analysis

Statistical analyses were conducted using R programming language [33].

To look for associations between the count of positive samples of each type compared with each other type, we performed pair-wise Kendall’s rank correlation tests, corrected for multiple comparisons with the Bonferroni-Holm adjustment. Correlations between quantity of IAV RNA copies between samples of oral fluids, surface and air inside the barns were also computed using Kendall’s correlation. Correlations of these samples with clinical scores were computed as well. Correlations between quantity of IAV in indoor air with recorded measurements of relative humidity and temperature were also determined.

To compute correlations and modeling of IAV in indoor air, data from four investigations with at least 5 days of samples were used (investigations 5, 9, 10 and 11). Data were limited to the first 21 days after the reported onset given that most of the farms tested negative after that, and the mean indoor air IAV quantity was calculated for each visit. The concentration of IAV in the air inside the barn as a function of time over the outbreak was modeled using a quasipoisson model with log link to appropriately handle both the days at which zeros were recorded and the fact that the variance increased with the quantity of virus detected. Additionally, as the reported day of onset may have been early or late relative to the true progress of the infection, the reported day of onset was allowed to shift relative to the estimated maximum for each investigation to minimize the deviance of the fit.

A quadratic effect was used for day and an additive blocking effect was used to allow each investigation to have a different maximum value; the fitted equation was:

Indoor air quantity = M*exp(-0.035*day—0.082*day^2), where M is the maximum value for that investigation, and day is relative to the day of the maximum.

## Results

### Clinical signs

Clinical scores of coughing and sneezing were recorded in both IAV positive and negative investigations. Mean scores ranged from 0.83% to 36.71% and 0.33% to 10.27% for sneezing and coughing, respectively (Table 2) and there was variation in the scores throughout the course of the investigations (results not shown).

**Table 2:** Average clinical scores of sneezing and coughing for each investigation.

Investigation ID	Sneezing score	Coughing score
1	12.00%	2.00%
2	3.33%	4.83%
3	5.65%	0.33%
4	4.80%	8.10%
5	5.25%	4.66%
6	0.83%	5.00%
7	7.41%	7.41%
8	28.02%	1.35%
9	36.71%	3.28%
10	28.80%	7.24%
11	26.55%	10.27%

### Influenza infections

Eleven suspected IAV outbreak investigations in barns corresponding to 6 farms were identified during the study. There were a total of 49 farm visits, which took place between 2 to 8 days apart for 4 to 42 days after the initial visit. Six of the 11 barn investigations, corresponding to three different farms, were confirmed positive for IAV by RRT-PCR testing in aerosols, surfaces, and/or swine oral fluid samples.

Forty-seven out of 98 (48%) oral fluid samples tested were RRT-PCR positive for IAV while 32 of 84 (38%) pen railing samples, and 35 of 82 (43%) indoor air samples tested positive for IAV (Table 3). There were two door handle samples that tested positive at low levels. All air samples collected outdoors tested negative. There was a significant positive correlation of 0.69 between the count of oral fluid positive samples and air (p = 0.0001), of 0.47 between oral fluids and pen railing (p = 0.009) and 0.42 between indoor air and pen railing (p = 0.01).

**Table 3:** Total number of samples positive for influenza A virus real time reverse transcriptase polymerase chain reaction (RRT-PCR), per sample type and investigation.

Investigation ID	Oral fluids	Air indoor	Pen railing	Door	Air downwind	Air upwind	Site influenza status
1	2/4*	0/2	0/4	0/1	0/2	0/2	Pos
2	0/6	0/6	0/6	0/3	0/6	0/6	Neg
3	0/8	0/6	0/6	0/3	0/6	0/6	Neg
4	2/8	1/6	0/6	0/3	0/6	0/6	Pos
5	9/12	6/12	2/12	0/6	0/12	0/12	Pos
6	0/2	NT	NT	NT	NT	NT	Neg
7	0/2	NT	NT	NT	NT	NT	Neg
8	0/6	NT	NT	NT	NT	NT	Neg
9	10/14	10/14	10/14	1/7	0/14	0/14	Pos
10	8/16	5/16	9/16	1/16	0/12	0/12	Pos
11	16/20	13/20	11/20	0/10	0/16	0/16	Pos
Total (%)	47/98 (48)	35/82 (43)	32/84 (38)	2/49 (4)	0/74(0)	0/74(0)	6/11(54)

* Number of positive RRT-PCR/total number of samples; NT: not tested

IAV was isolated by culture from 19 oral fluid and 18 indoor air samples (Table 4) representing five and four investigations, respectively. H1N1, H1N2 and H3N2 subtypes, and mixtures of these, were identified in both, oral fluid and indoor air samples. Virus isolation of surface samples did not yield positive results.

**Table 4:** Influenza A virus subtype and number of isolates cultured from oral fluids and indoor air samples in each confirmed positive investigation.

Investigation ID	No.isolates (oral fluids)	No.isolates (air indoor)	Subtype oral fluids	Subtype air
1	0	0	-	-
4	2	0	H1N2, H1N1	-
5	4	5	H1N1, H1N2	H1N2, H1N1
9	6	6	H1N1	H1N1
10	3	2	H3N2	H3N2
11	3	4	H1N1, H3N2	H1N1, H3N2
Total	19	18		

### Viral quantification

Influenza RNA levels in oral fluids, indoor air and pen railing varied between farms and throughout the course of the clinical outbreaks (Table 5). Individual sample viral levels ranged from 0 to 4.03x10^7^ RNA copies/ml in oral fluids, 0 to 4.16x10^7^ RNA copies/m^2^) in pen railing surface and 0 to 1.25x10^6^ RNA copies/m^3^ of air in indoor air samples. There was a significant positive correlation of 0.4 between quantity of IAV in the air and oral fluids (p = 0.015) and of 0.372 between quantity of IAV in oral fluids and coughing (p = 0.023) (Table 6). Correlations between quantity of IAV on the pen railing with both the air and oral fluids were not significant (p>0.05).

**Table 5:** Mean influenza A virus (IAV) quantitative real time reverse transcriptase polymerase chain reaction (RRT-PCR) results for oral fluids, pen railing and indoor air samples from IAV positive farms.

Investigation ID	Oral fluids (RNA copies/ml)	Pen railing (RNA copies/m^2^)	Indoor air (RNA copies/m^3^ air)
1	2.26E+05	0.00E+00	0.00E+00
4	6.26E+05	0.00E+00	9.48E+03
5	5.25E+06	3.58E+06	1.72E+05
9	5.08E+05	4.70E+06	2.44E+05
10	3.67E +06	3.29E+06	1.33E+05
11	1.55E+06	2.67E+06	1.32E+05
Mean	2.17E+06	1.42E+06	1.42E+05
Standard Deviation	6.89E+06	2.67E+06	2.67E+05

**Table 6:** Correlations of quantity of influenza A virus in oral fluids of groups of pigs and environment and with percentage of pigs exhibiting coughing, sneezing or signs of illness.

	Pen railing	Indoor air	Oral fluids
% Ill pigs	0.088	0.139	0.080
Coughing pigs	-0.084	0.124	0.372*
Sneezing pigs	0.073	0.119	-0.021
Indoor air	0.23	-	0.40*
Oral fluids	0.22	0.40*	-

* Differences p<0.05

### Environmental conditions

Measured mean indoor temperatures ranged between 19°C and 25°C while relative humidity ranged between 19°C and 25°C. Both of these ranges were within the expected ranges for swine commercial facilities. Correlations between quantity of IAV in indoor air samples with relative humidity and temperature were 0.26 (p = 0.12) and 0.01 (p = 0.93) respectively.

### Influenza level modeling

**Fig 1:**
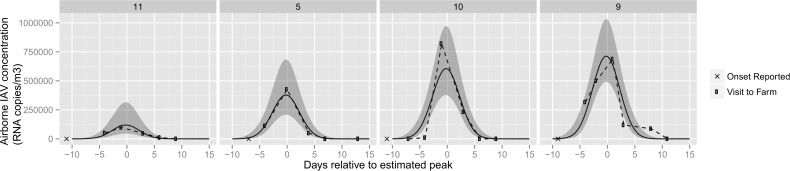
Modeled influenza A virus quantity (RNA copies/m^3^) in indoor air. Fig shows the data, connected by dashed lines, and the fitted values in a solid line, as well as 95% confidence interval for the fitted values. Axis x represents the days relative to the estimated peak of the outbreak. The date when the onset was reported is also shown.

Results of modeling of indoor air levels of IAV are shown in Fig 1 based on results from 4 investigations. The best fit for the reported day of onset relative to the estimated maximum was -11, -11, -7 and -9 days for investigations 10, 11, 5 and 9, respectively. These results indicated a short spread between farms in the duration of IAV detection in the air. The model also showed the best fit for the estimated mean maximum RNA copy viral load (x10^4 RNA copies/m^3^) with 95% confidence interval for each investigation at 12 (4.5, 31), 38 (21, 68), 60 (38, 97), and 71 (49, 103) for investigations 11, 5, 10, and 9 respectively, indicating differences in the modeled levels of airborne IAV between farms and throughout the duration of an outbreak. Data from oral fluids and surfaces could not be modeled because of a lack of a common pattern in the data obtained.

## Discussion

Despite the common occurrence of IAV infections in swine, there is limited information on the levels and persistence of IAV in the air and environment of swine production facilities. This is the first study, to our knowledge, that has quantified and characterized the level of IAV in samples of aerosols and surfaces of swine environments during acute outbreaks of influenza infections in swine. We found that IAV could be isolated from indoor air of commercial swine production facilities, that airborne IAV levels were sustained for periods of 20 days and that there was a correlation between the number of positive samples of each type and the quantity of virus in the swine oral fluids and in the air. Our results provide a first estimation on levels of environmental IAV in swine commercial production facilities, and thus an assessment of potential sources of IAV exposure to swine workers or other pigs.

Detection of IAV in air was sustained throughout the course of the acute outbreaks and lasted approximately 20 days across the studied barns. The peak of detection of virus in air samples occurred between 7 and 11 days into the outbreaks. Maximum airborne levels varied between affected facilities and were in the order magnitude of 10^4^ to 10^7^ RNA copies/m^3^ of air. We allowed our model to shift relative to the estimated maximum for each investigation given that the reported day may not have been when the infection truly started. Interestingly the IAV detection curves were similar across farms with limited variations in duration which suggests a similar course of disease between farms and, that similar measures could be implemented to minimize risk of IAV infections. There were differences in the levels of IAV found in the air between farms and we speculate that these differences could be due to varying levels of infection and immunity in the swine, type of IAV, number of pigs in the barn, barn volume, ventilation rates and farm and management characteristics. Thus, information from our model can be used to estimate risk of exposure to swine workers, other pigs and help target intervention strategies to mitigate the risk of IAV transmission between pigs and from pigs to people.

A key aspect of determining the risk of IAV transmission is the relationship between number of RNA copies and infectivity. We estimated the ratio of viral particles to TCID_50_ (tissue culture infectious dose) at 3,000 RNA copies/TCID (results not shown) and based on the mean airborne IAV concentration, our results corresponded to 47 TCID_50_/m^3^. Similar TCID_50_ estimates were obtained in a health center [34] and although it is unclear how our results relate to transmission to swine or people, we speculate that they represent a significant risk to both people and swine, since IAV was readily isolated from the air multiple times throughout the duration of the outbreaks. Overall, our results provide evidence that air can be an important route of IAV transmission in swine production facilities. Furthermore there was an association between the levels of IAV in oral fluids and the air indicating a direct relationship between level of virus in the swine and potential exposure through aerosols. However, further studies are needed to fully understand the relationship between airborne IAV levels and transmission.

We isolated a mixture of genetically diverse IAV from swine and air samples representing the three most common IAV subtypes in swine, H1N1, H1N2 and H3N2. However, in contrast to a prior study [23], we did not detect IAV in air samples collected outside swine facilities. This difference is probably a result of sampling frequency and our investigations being carried out under colder environmental conditions and larger distances from the air exhaust site that likely negatively impacted both distribution of the virus and viral survivability. Therefore more research is needed to fully characterize the risk of IAV transmission outside swine production facilities.

IAV genetic material was also detected in surfaces, in particular on pen railings although we could not isolate IAV from surfaces. Source of IAV genetic material in the surfaces may be the result of deposition of airborne IAV particles. Whether inability to culture IAV from surfaces was due to lack of viable IAV in surfaces or conditions of sampling or limited sensitivity of the culture technique could not be assessed, but we speculate that viable IAV can still be present on surfaces from swine barns although with less quantity than in the air due to environmental conditions such as desiccation or preservation in dust. In deed surface contamination with viable IAV was shown in a live animal market housing swine [3]. Therefore, precautions to prevent exposure to contaminated surfaces should still be followed.

Clinical signs of coughing and sneezing can be an indicator of IAV infections and IAV can be found in both clinically and subclinically infected swine [2]. We found an association between coughing and levels of IAV in the swine but not in air or on surfaces. We did not have a common pattern on the presentation and evolution of clinical signs across farms. This could be due to presence of concomitant infections or farm factors not measured in this study. Overall our results indicate that clinical signs in swine cannot be used as a reliable indicator of the levels of IAV present in the environment and thus they should not be used to predict risk of exposure to people.

The farm investigations in this study were selected by convenience based on recognized acute clinical signs in the swine herds, thus results from this study should be interpreted carefully when extrapolating them to endemically infected farms. Furthermore data in this study was obtained from a limited number of farms which do not represent the full spectrum of types of production facilities and management conditions encountered in the swine industry. Thus further research is needed to characterize the levels and risk of IAV environmental exposure in non-outbreak situations and production facilities representative of different management and environmental conditions.

Lastly, although environmental conditions of relative humidity and temperature have been associated with IAV viability [35], in this study we did not see an association between quantity of IAV in air or on surfaces and relative humidity or temperature. This lack of association could be the result of the relatively stable indoor conditions throughout our study. Producers put a great deal of effort into maintaining a relatively stable temperature and relative humidity within these facilities to maintain the health and safety of the animals.

In summary, our results indicate that during outbreaks of IAV in swine, the air and surfaces in barns contain significant levels of IAV potentially representing an exposure hazard to both swine and people. Further studies are needed to evaluate the viability of IAV in the environment, evaluate strategies to mitigate the risk of indirect transmission of IAV, confirm the impact of personal protective equipment on exposure risk to people and explore strategies to prevent bidirectional transmission of IAV between humans and swine. Information from this study should help to develop evidence-based guidelines to minimize the impact of IAV infections on swine production.
